# Genomic, microbial and environmental standardization in animal experimentation limiting immunological discovery

**DOI:** 10.1186/s12865-020-00380-x

**Published:** 2020-09-02

**Authors:** Josue Enriquez, Brianyell Mc Daniel Mims, Scott Trasti, Kathryn L. Furr, Matthew B. Grisham

**Affiliations:** 1 Department of Immunology and Molecular Microbiology, Texas Tech University Health Sciences Center, 3601 4th Street STOP 6591, Lubbock, TX 79430-6591 USA; 2 Laboratory Animal Research Center, Texas Tech University Health Sciences Center, Lubbock, TX 79430 USA

**Keywords:** Outbred mice, Infection, Inflammation, Microbiota, Obesity, GVHD, Steatohepatitis, Atherosclerosis, Autoimmune disease, Colorectal cancer

## Abstract

**Background:**

The use of inbred mice housed under standardized environmental conditions has been critical in identifying immuno-pathological mechanisms in different infectious and inflammatory diseases as well as revealing new therapeutic targets for clinical trials. Unfortunately, only a small percentage of preclinical intervention studies using well-defined mouse models of disease have progressed to clinically-effective treatments in patients. The reasons for this lack of bench-to-bedside transition are not completely understood; however, emerging data suggest that genetic diversity and housing environment may greatly influence muring immunity and inflammation.

**Results:**

Accumulating evidence suggests that certain immune responses and/or disease phenotypes observed in inbred mice may be quite different than those observed in their outbred counterparts. These differences have been thought to contribute to differing immune responses to foreign and/or auto-antigens in mice vs. humans. There is also a growing literature demonstrating that mice housed under specific pathogen free conditions possess an immature immune system that remarkably affects their ability to respond to pathogens and/or inflammation when compared with mice exposed to a more diverse spectrum of microorganisms. Furthermore, recent studies demonstrate that mice develop chronic cold stress when housed at standard animal care facility temperatures (i.e. 22–24 °C). These temperatures have been shown alter immune responses to foreign and auto-antigens when compared with mice housed at their thermo-neutral body temperature of 30–32 °C.

**Conclusions:**

Exposure of genetically diverse mice to a spectrum of environmentally-relevant microorganisms at housing temperatures that approximate their thermo-neutral zone may improve the chances of identifying new and more potent therapeutics to treat infectious and inflammatory diseases.

## Background

All FDA-approved drugs that are currently in use clinically in the U.S. were developed and tested in animal models of disease so that drug toxicity and safety, dose–responses and efficacy could be determined. Because mice share many of the same anatomical, physiological, immunological and genetic properties with humans, these small mammals have become the primary animal of choice for modeling human infectious and inflammatory diseases [1]. Indeed, the use of inbred mice housed under standardized environmental conditions has been crucial to our understanding of the immuno-pathological mechanisms involved in different mouse models of disease as well as identifying therapeutic targets that are tested in clinical studies. Furthermore, the use of genetically standardized mice exposed to a well-defined, pathogen free microbiota has markedly reduced the variability of disease phenotype reported by different laboratories. Despite these important advancements, only 10% of promising therapeutic strategies reported in mouse models of disease have gone on to be used as clinically-effective treatments in patients [2–4]. It has been suggested that differences in murine vs. human immune systems may account for the reduced success of translation of preclinical data [3, 5–11]. It is well-known that the structure and cellular composition of the murine immune system is, in some cases, substantially different than that of humans [6, 12–16]. These differences are thought to contribute to differing immune responses to foreign and/or self-antigens in mice vs. humans [17]. Indeed, the debate continues as to whether genomic responses of mice during inflammation mimic those in humans [18–20]. In addition to differences in immunological responses between the two species, there is a growing literature suggesting that housing inbred mice in ultra-clean conditions at temperatures well-below their thermoneutral zone, may significantly alter their response to infectious micro-organisms and tumor cells as wells as to allo- and auto-antigens. For example, inbred mice housed under specific pathogen free (SPF)/barrier conditions may respond to microbial infections or inflammatory mediators differently than do outbred mice or inbred mice colonized with more diverse communities of microorganisms [12, 15]. In addition, virtually all animal care facilities house mice at 22–24 °C that is well below their thermoneutral zone. These housing temperatures create chronic cold stress that are known to alter murine immune responses to different foreign or auto antigens when compared with mice housed at their thermo-neutral body temperature of 30–32 °C [15, 21–24]. When taken together, it is reasonable to assume that these intrinsic and extrinsic factors may markedly affect the translational nature of preclinical studies using mouse models of infectious and inflammatory diseases. This review discusses how genetic diversity and the environment affects immunity and inflammation in mice and describes how potential modifications of current animal husbandry and experimental protocols may help to more closely model infectious and inflammatory diseases.

### Murine genetic diversity

The use of mice to study immune responses to infection and inflammation have revealed a number of fundamental principles that have been confirmed in humans. One paradigm-changing example of how mouse models have advanced our understanding of immune regulation is recognition that failure to maintain tolerance to commensal bacteria and/or autoantigens results in the development of chronic inflammatory diseases [25–27]. Although these and other important studies have helped to uncover a number of novel immuno-pathogenic mechanisms underlying infectious and inflammatory diseases, relatively few of the promising therapeutic treatments or interventions reported in follow up studies have been translated into effective therapies in humans [2–4, 7, 17]. As discussed above, several reasons have been proposed to account for the low bench-to-bedside transition that have largely focused on differences in immune system composition and function in mice vs. humans. More recent studies have begun to address the likelihood that the restricted genetic diversity of inbred mice may limit their ability to express the full range of immune responses that are observed in diverse human populations. Accumulating evidence suggests that certain immune responses and/or disease phenotypes observed in inbred mice may be quite different than those observed in their outbred counterparts [28–32]. This is not surprising given the fact that inbred strains of mice are produced by single lineage brother/sister mating for at least 20 generations. Because this type of breeding scheme produces mice that are genetically identical, there is far less inter-animal variability. However, it should be noted that this type of breeding scheme produces inbreeding depression and reduced genetic diversity [33]. The use of inbred mice to model human disease is tantamount to using “multiple copies of one individual” [17]. While this tool has been crucial to our understanding of innate and adaptive immunity, it most likely does not capture the hybrid vigor and genetic diversity present in humans.

Historically, outbred mice have been used for toxicological, pharmacological, cancer and aging research. The most common outbred stocks used in preclinical studies include CD-1, Institute for Cancer Research (ICR), Swiss Webster (SW) and NIH Swiss mice [33]. The majority of outbred mice used in the U.S. today are derived from 2 male and 7 female, non-inbred albino mice that were imported from Switzerland by Dr. Carla Lynch to the Rockefeller Institute for Medical Research in 1926 [34]. These original stocks were collectively referred to as “Swiss” mice. It should be noted that different lineages of outbred mice are referred to as “stocks” whereas inbred lineages of mice are referred to as “strains” [33]. By definition, outbred stocks are “a closed population … of genetically variable animals that are bred to maintain maximum heterozygosity” [34]. In theory, no two individuals are genetically identical within an outbred stock. Siblings of inbred strains of mice are genetically identical that are maintained through brother and sister mating for 20 or more generations [33]. Because outbred stocks are genetically more diverse than inbred strains, data obtained using these mice tend to be more variable requiring substantially larger numbers of animals for statistical power [34]. Thus, the majority of published studies exploring the immuno-pathogenesis of infectious and inflammatory diseases have used the more genetically homogeneous inbred strains. Despite the advantages of using inbred strains, there is a clear need to understand how genetic diversity influences immunity and inflammation. Below, we present an overview of several studies that highlight the differences in immune responses between different strains and stocks of mice subjected to infection or inflammation.

#### Use of outbred stocks to study infectious diseases

It is now well-known that the susceptibility of inbred and outbred mice to infectious micro-organisms may be quite different. Thus, translation of preclinical data based upon only one strain or stock may be problematic. For example, *Citrobacter rodentium* (*C. rodentium*) infection induces severe but self-limiting diarrhea, colonic epithelial hyperplasia and mild intestinal inflammation in a variety of mice on different genetic backgrounds. Borenstein et al. demonstrated that infection of outbred SW mice with *C. rodentium* recapitulated this phenotype i.e. subclinical and self-limiting intestinal inflammation [35]. However, when inbred mice derived from the SW stock [called Friend Virus B (FVB)] mice were infected with *C. rodentium*, they developed a lethal disease characterized by severe ulcerative colitis [35]. In a more recent study, Sunagar et al. compared the immune responses of SW vs. inbred C57BL/6 mice to infection with the highly virulent pathogen *Franscisella tularensis* (Ft) [36]. This Tier 1 bio-threat has been extensively studied over the past several years yet no FDA-approved vaccine has been developed. Sunagar et al. found that outbred SW mice were more resistant to Ft challenge and were better protected from the lethal effects of this pathogen following vaccination than were inbred C57BL/6 mice [36]. The authors concluded that outbred mice may more accurately reflect the genetic diversity of human immunity to this deadly pathogen. Another example of differential susceptibility between inbred and outbred mice to an infectious agent was recently reported by Carreras et al [37].. These investigators found that C57BL/6 mice were more susceptible than CD1 mice to the lethal effects of sepsis induced by intravenous administration of the fungus *Candida albicans* when compared with C57BL/6 [37]. In another study using a mouse model of *Streptococcus suis (S. suis*)-induced sepsis and cerebral inflammation, Dominguez-Punaro et al. reported that while infection of different inbred strains of mice (e.g. BALB/c, C57BL/6, A/J) with *S. suis* caused septic shock and death, it did not induce meningitis and encephalitis [38]. In contrast, these investigators showed that infection of outbred CD1 mice with *S. suis* induced rapid and systemic production of high levels of several different inflammatory cytokines that likely contributed to the death of 20% of the mice within the first 48 h following infection [39]. In those mice that survived, approximately 40% developed clinical signs of meningitis between days 4 and 9 post-infection. These exciting studies provided the first demonstration that hematogenous infection with *S. suis* produces both septic shock and cerebral inflammation.

One immune cell that plays a critical role in recognizing and eliminating intracellular pathogens (e.g. viruses, bacteria, protozoan) and tumor cells is the CD8^+^ T cell [40]. The ability to accurately define the magnitude and kinetics as well as the phenotypic and functional characteristics of CD8 T cells following pathogen infection is not only important for our understanding of immune regulation but it is also crucial for developing new and more robust vaccination protocols. Much of what we have learned about T cell-mediated immunity has come from studies using inbred mice [31, 41]. Recent studies from the Badovinac laboratory have shown that infection of outbred SW mice with either *Listeria monocytogenes (LM)* or Armstrong strain of lymphocytic choriomeningitis virus (LCMV) results in large animal-to-animal variations in pathogen-specific CD8 T cell responses when compared with their inbred C57BL/6 counterparts. These data suggest that genetic background may control the magnitude of CD8 T cell expansion in response to pathogen infection [31, 41]. In addition, Martin et.al. found that infection of SW mice with LM or LCMV generated a number of different populations of pathogen-specific CD8 T cells and much greater variability in the kinetics of phenotypic progression of Ag-specific CD8 T cells when compared with C57BL/6 mice [31]. Importantly, these investigators found, using an infection/reinfection protocol, that C57BL/6 mice were protected from the lethal effects of LM whereas survival of SW mice was significantly more variable. These studies illustrate the larger degree of variability of T cell responses to pathogen infection in outbred mice.

#### Use of outbred stocks to study inflammatory diseases

##### Allograft rejection

The use of genetically restricted, inbred mice have been the mainstay for the large majority of studies exploring the immunopathogenesis of transplant rejection a variety of different tissue allografts. In one of the few studies that has assessed the effects of genetic diversity of donor and recipient on allograft survival in mice, Reichenbach et al. performed vascularized, heterotopic heart transplantation in outbred and inbred mice [29]. They found that all heart allografts that were obtained from an inbred strain and transplanted into a different recipient strain (e.g. inbred→inbred) or into an outbred stock (inbred→outbred) were rejected within 6–16 days. In contrast, when heart allografts obtained from outbred donors and transplanted into an inbred (outbred→inbred) or outbred recipient (outbred→outbred), they observed a spectrum of outcomes that included very early rejection (< 4 days) in 30% of the recipients, acute rejection (6–24 days) in 54% of the transplanted recipients and chronic rejection (> 75 days) in 17% of the recipients [29]. The striking, very early rejection observed in 30% of the outbred→inbred and outbred→outbred mice did not appear to be classic hyperacute rejection since the presence of preformed, anti-donor antibodies in recipients was very low or absent. In fact, very early allograft rejection was characterized by neutrophilic vasculitis and hemorrhagic necrosis that could be abrogated by depletion of neutrophils or complement [29]. These investigators concluded that very early allograft failure was dependent on the genetic diversity of outbred donor mice and not the recipient.

##### Intestinal inflammation

A number of mouse models of the inflammatory bowel diseases (e.g. IBD; Crohn’s disease, ulcerative colitis) have been developed to investigate the immuno-pathogenesis of these chronic inflammatory disorders. Although a great deal of important mechanistic information has been obtained using these preclinical mouse models, only a small percentage (< 10%) of promising targets and therapeutic interventions have been translated to patient treatment [3, 7]. A recent study by Barone et al. sought to determine how genetic diversity (and gender) affects the onset and severity of chronic colonic inflammation induced by intra-rectal administration of the haptenating agent dinitrobenzene sulfonic acid (DNBS) in C57BL/6 inbred vs CD1 outbred male and female mice [32]. Colonic inflammation was induced via two intra-rectal injections of DNBS spaced 21 days apart and assessed for evidence of colonic inflammation. Barone et al. found that colonic administration of DNBS significantly reduced survival of CD1 male and female mice compared with C57BL/6 mice as well as reduced body weight to a greater extent in CD1 vs. C57BL/6 mice. In addition, CD1 mice displayed greater macroscopic and histopathological evidence of colitis when compared with their inbred counterparts, suggesting that CD1 outbred mice may be more susceptible to chemically-induced colitis [32]. These findings suggest that the use of outbred mice may provide investigators with new information to better understand the immunomodulatory mechanisms associated with this model of chronic colitis.

##### Inflammatory angiogenesis

Inflammation and angiogenesis are important physiological responses that are required for fibrovascular tissue growth and wound healing. However, dysregulation of this proliferative response may result in the development of chronic inflammatory diseases such as atherosclerosis, rheumatoid arthritis, asthma and inflammatory bowel disease, to name just a few [42–47]. Recent studies by Andrade and coworkers examined how host genetic diversity may influence the development and perpetuation of inflammatory angiogenesis and fibrogenesis. Using a well-characterized mouse model of peritonitis, they compared the immuno-pathological responses (e.g. inflammatory cell infiltration, angiogenesis and fibrosis) in three inbred strains (DBA-1, BALB/c, C57BL/6) and one outbred strain (Swiss) [47, 48]. These investigators observed highly variable responses among the four different groups of mice at 7 days following implantation. For example, angiogenesis and macrophage infiltration were greatest in C57BL/6 mice when compared with the other three inbred strains of mice [47]. Interestingly, fibrogenesis markers, as assessed by implant-derived transforming growth factor beta-1 and collagen were consistently lower in DBA/1 mice when compared with the other three inbred strains. In addition to defining the angiogenic and fibrogenic profiles among the different inbred and outbred mice, Marques et al. investigated how host genetics affected the anti-platelet activity of dipyridamole (DP) in their peritonitis model [47]. Overall, they found that DP treatment exerted a more generalized anti-inflammatory response in inbred strains vs outbred Swiss mice with significant anti-fibrogenic effects observed only in C57BL/6 mice [47]. Taken together, these data demonstrate that inflammatory angiogenesis is highly strain dependent and highlight the importance of host genetics in these models of peritonitis.

##### Neuroinflammation

Murine experimental autoimmune encephalomyelitis (EAE) is a mouse model of T cell-mediated demyelinating disease of the central nervous system (CNS) that is used to study the immune-pathogenesis of human multiple sclerosis (MS) [49, 50]. This autoimmune model of MS is induced in mice via immunization with different myelin-derived antigens such as myelin basic protein (MBP), myelin oligodentrocyte glycoprotein (MOG) or proteolipid protein (PLP) [49, 50]. It is well-recognized that different inbred strains of mice exhibit differential susceptibility to antigen-induced EAE [49, 50]. One of the few published studies that have compared the immunological responses of susceptible C57BL/6 mice to those of resistant *outbred* CD1 mice following immunization with the MOG_35–55_ peptide has been reported by Marin et al. [51]. Using identical immunization protocols, these investigators found that T cells obtained from immunized C57BL/6 mice were capable of enhanced proliferation when challenged with MOG_35–55_ in vitro whereas T cells from CD1 mice showed little or no proliferation. In addition, Marin and coworkers found that immunization of CD1 mice with MOG_35–55_ peptide increased the percentages of regulatory T and B cells when compared with C57BL/6 mice. The authors concluded that the resistance of CD1 mice to MOG_35–55_ induced EAE may be mediated by expansion of regulatory T and B cells via MHC-independent mechanisms.

Nikodemova et al. compared neuro-inflammatory responses of microglial cells obtained from inbred C57BL/6 vs. outbred ICR/CD1 mice subjected to lipopolysaccharide (LPS) administration in vitro and in vivo. For their in vitro studies, they used BV2 and N9 microglial cell lines that were originally derived from either C57BL/6 inbred mice or ICR/CD1 outbred mice, respectively as well as primary microglial cells derived from the brains of the two different mice [28]. They found that addition of LPS to N9 cells (derived from ICR/CD1 mice) induced large and significant production of TNFα and the nitric oxide (NO)-derived metabolite, nitrite that were significantly greater than those produced by the LPS-activated BV2 (C57BL/6) microglial cells [28]. In addition, they found that addition of LPS to ICR/CD1 primary microglial cells induced greater amounts of TNFα and nitrite when compared with LPS-activated microglial cells obtained from C57BL/6 mice. Furthermore, intra-parenchymal injection of LPS induced greater expression of mRNA for TNFα, IL-6 and inducible NO synthase in microglial cells from ICR/CD1 vs C57BL/6 mice [28]. Taken together, these data demonstrate the influence of host genetics on microglial responses to inflammatory mediators in vitro *and* in vivo.

#### Development, characterization and use of genetically diverse collaborative Cross mice

**Fig. 1:**
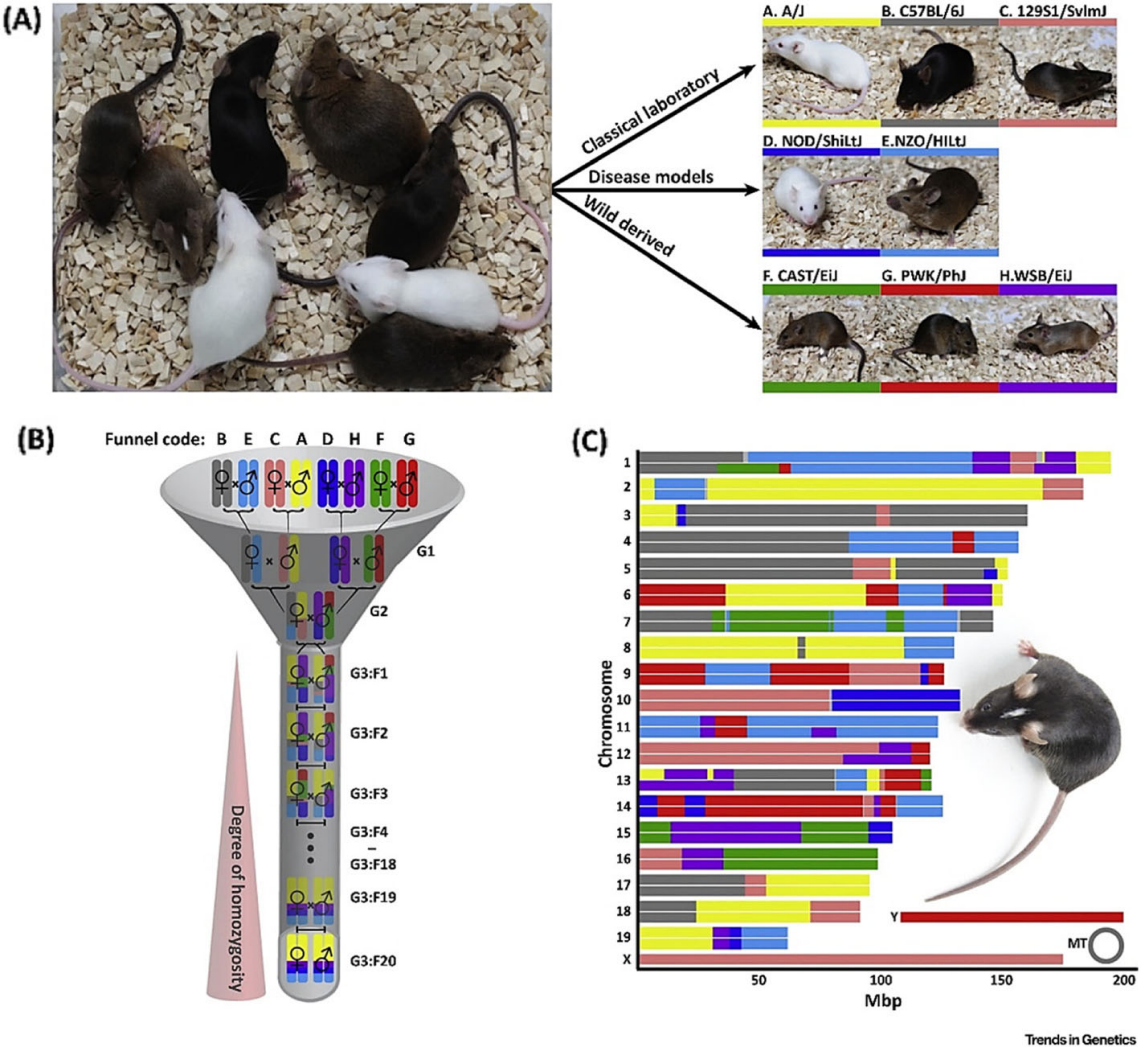
Generation of Collaborative Cross mice. **a.** This unique panel of genetically diverse mice was generated by interbreeding 8 unique and genetically diverse founder strains of mice that included *3 inbred laboratory strains* (C57BL/6, A/J, 129S1/SvImJ), *2 inbred mouse models of disease* (NOD/ShiLtJ-*type 1 diabetes* and New Zealand-*obese/type 2 diabetes* mice) and *3 wild*-derived strains (CAST/EiJ, PWK/PhJ, and Watkins star line B (WSB)/EiJ mice). Each strain was assigned both a letter (A–H) and a specific color. **b** A combinational funnel breeding scheme was used to produce offspring with equal distribution of founder alleles. This figure illustrates the specific breeding funnel for the generation of the CC strain CC001. **c** Color illustration of founder genome contributions to CC001 strain. Reproduced from [54] with permission

The use of the commercially available stocks of outbred mice has provided investigators with a greater understanding of range and variability of immune responses that may not be observed using inbred strains. Nevertheless, outbred stocks may in many cases, exhibit genetic lability as well as a poorly defined genotype and genetic variation within and between different stocks [34]. In an attempt to approximate the complex interactions of genes involved in human immune responses, a genetically-diverse reference population of mice has been generated that is referred to as Collaborative Cross (CC) mice [52, 53]. This unique panel of genetically diverse mice was generated by combinational “funnel” breeding schemes using 8 unique and genetically diverse founder strains of mice that included *3 inbred strains* (C57BL/6, A/J, 129S1/SvImJ), *2 inbred mouse models of disease* (NOD/ShiLtJ-type 1 diabetes and New Zealand-obese/type 2 diabetes mice) and *3 wild*-derived strains (CAST/EiJ, PWK/PhJ, and Watkins star line B (WSB)/EiJ mice) (Fig. 1) [30, 53–55]. Partial recombinant inbred strains of CC mice (termed pre-CC mice) were produced by three generations of funnel breeding followed by brother-sister/inbred mating for 4–5 generations. Over the past few years, different strains of the pre-CC mice were interbred for at least 20 generations thereby creating numerous recombinant inbred CC strains termed CC-RI strains. Both pre-CC and CC-RI strains contain genetic contributions from all eight of the original founders that display ~ 90% of the known genetic variation present in wild mice (*Mus musculus*) [30, 53]. Each mouse within a given CC-RI strain is genetically identical containing maximum allelic variation whereas each CC-RI strain is genetically distinct from all other CC-RI strains. To date, more than 60 individual CC-RI strains have been created at the University of North Carolina (http://www.csbio.unc.edu/CCstatus/index.py?run=CCV). Additional CC strains of mice have been created by interbreeding two different CC-RI strains to generate F1 recombinant intercross offspring (CC-RIX) that are heterozygous for the H-2b major histocompatibility complex (MHC) [30, 56]. This type of breeding scheme allows investigators to use specific reagents (e.g. MHC tetramers) to examine different aspects of T cell-mediated immunity. Pre-CC, CC-RI and CC-RIX mice have been used to characterize immune responses to human viral pathogens (e.g. influenza, West Nile and Ebola), chronic inflammation and cancer [30] (and references therein). By observing the spectrum of disease phenotypes in models of infectious and inflammatory diseases using well-defined strains of CC-RI or CC-RIX strains in which the genetics have been well-characterized, investigators may be able to identify *disease susceptibility loci* [54]. Multiple CC strains have been used to identify promising candidate genes that influence susceptibility or resistance to viral infections [54].

The studies outlined above provide examples of how murine genetic diversity may affect immune responses to pathogens and/or inflammatory mediators. These studies suggest that inbred strains may be better suited for initial investigations to define specific immunopathological mechanisms of infectious and inflammatory diseases with follow up studies using outbred mice to address the role of genetic diversity in these models.

### Microbial diversity and function

The mammalian body is home to a plethora of microorganisms that include prokaryotes (bacteria and archaea), viruses and eukaryotes (fungi, helminths and protozoa). A recent reevaluation of the total numbers of bacteria in the human body reports that the colon contains 10^14^ bacteria followed by the small bowel (10^11^), skin (10^11^) and saliva (10^11^) [57, 58]. Under normal, steady state conditions, these commensals play a critical role in the development, instruction and function of the host immune system [59]. Although the host immune system limits translocation of commensal bacteria into tissue and the systemic circulation, emerging evidence demonstrates that bacterial components and metabolites gain access to the systemic circulation and peripheral tissues where they may modulate tissue function [60]. Josefsdottir et al. recently reported that intestinal bacterial products such as microbial-associated molecular patterns are required for robust hematopoiesis [61] Other groups of commensal microorganisms that are particularly prevalent in the gut lumen are eukaryote and bacterial viruses [62]. In addition to their well-known pathogenic properties, both groups of viruses are known to possess potent immunomodulatory properties. For example, studies have shown that infection of mice with certain eurkaryote viruses are capable of attenuating development of chronic inflammation in mouse models of Type I diabetes and SLE-like disease (see [62] and references therein). In addition, Liu et al. recently reported that eukaryotic viruses are required for maintaining appropriate numbers of intraepithelial lymphocytes in the small and large bowel [63]. Bacterial viruses (i.e. bacteriophage) are also thought to protect the gut and host by infecting and killing pathogenic bacteria. A study by Barr et.al. demonstrated that bacteriophage are highly enriched in the gut mucus layer where they are thought to interfere with adhesion of pathogenic bacteria to the gut epithelium thereby preventing or limiting their invasion into the tissue [64]. The mammalian gut and skin are also colonized with a diverse population of fungi. Although not as well studied as other commensals, data suggest that certain fungi play important roles in immune system development. Zhang et al. reported that intra-gastric administration of *Candida tropicalis (but not Trichosporon asahii* nor *Saccharomyces cerevisiae)* to immune cell- deficient germ free mice or wild type mice treated with an antifungal cocktail promoted the migration of retinaldehyde dehydrogenase positive dendritic cells into lymph nodes (LNs) draining the gut and skin thereby stimulating the development of functional LNs and gut-associated lymphoid tissue [65]. In another study, Markey et al. reported that colonization of mice with *Candida albicans* protects mice from a subsequent lethal challenge with the bacterial pathogen *Clostridium difficile* [66]*.*

Although the vast majority of all microbiome studies have focused on prokaryotes, emerging studies are demonstrating multi-cellular helminths and unicellular protozoa represent additional groups of commensal residents in the healthy gut that possess immunomodulatory activity. Historically, past investigations have focused almost exclusively on the parasitic and pathogenic properties of these eukaryotes. Although these multicellular organisms can produce severe disease, colonization does not necessarily result in disease [67] Recent studies suggest that these organisms may in fact, represent true commensal microbiota that may possibly protect their host [68, 69]. For example, helminths are known to release a variety of excretory-secretory (ES) components that have been shown to modulate the functions of several immune cells as well as induce the generation of immunosuppressive regulatory T cells (Tregs) and modify the intestinal microbiota (reviewed in [68]. Indeed, there is a growing literature demonstrating that colonization of animals with helminths or treatment with certain ES components attenuate allergic and chronic inflammatory diseases [68, 70]. In addition to helmiths, the parasitic and disease producing properties of protozoa have been studied extensively over the past several decades. Recent evidence suggests that while many protozoa are indeed pathogens, several of these unicellular microorganisms have been identified in the healthy microbiota [69]. One such commensal is *Blastocystis spp*. This protozoa has been shown to enhance the diversity and alter the microbial composition of individuals in industrialized countries, suggesting that microorganisms may exert a beneficial effect to the host [69]. These observations are particularly important given the fact that industrialized countries have attempted to eliminate both helminths and protozoa thereby limiting parasitic infections. Loss of these and possibly other commensal eukaryotes appears to be associated with dramatic increases in allergic and inflammatory diseases over the past five decades [68, 69].

A major breakthrough in our fundamental understanding of innate and adaptive immunity occurred following the generation of mice containing spontaneous or genetically-engineered alterations in specific genes related to immune system development and/or function. Because many of these mutant mice were immuno-compromised, it was necessary to develop animal housing conditions that would prevent the introduction of pathogens into the mouse colonies. In addition to maintaining a healthy environment for these mice, eliminating the introduction of known mouse pathogens into the vivarium allowed for more consistent breeding of mice as well as more reproducible induction disease phenotypes [71]. Although the term “specific pathogen-free” (SPF) was first used in the 1950s, it wasn’t until the 1980s when animal care facilities began to use filter top micro-isolator cages in conjunction with meticulous biocontainment protocols to limit the introduction of murine pathogens into these facilities [71]. Currently, all animal care facilities in the U.S. continuously monitor for (and exclude) known murine pathogenic viruses, bacteria and parasites to create SPF conditions. In addition, mice are housed under *barrier* conditions that require animals to be kept in micro-isolator cages with filter top lids or in individually ventilated cages (IVC) that use HEPA filtered air rather than ambient air. Furthermore, barrier facilities use sterilized cages, food, and water. These ultra-hygienic housing conditions have been incredibly important for the biomedical research community because they essentially “standardized” the mouse microbiome that appeared to reduce the variability of results observed by different laboratories. However, recent evidence suggests the immune system of mice raised under SPF/barrier conditions is immature when compared with their *wild* (i.e. feral) counterparts and to adult humans suggesting natural exposure to pathogens may be important in promoting maturation of the immune system [12, 16, 71]. The following sections will focus on how the composition of intestinal bacteria influences immunity and inflammation.

#### Modulation of immunity and inflammation by the microbiome

##### Infection

**Fig. 2:**
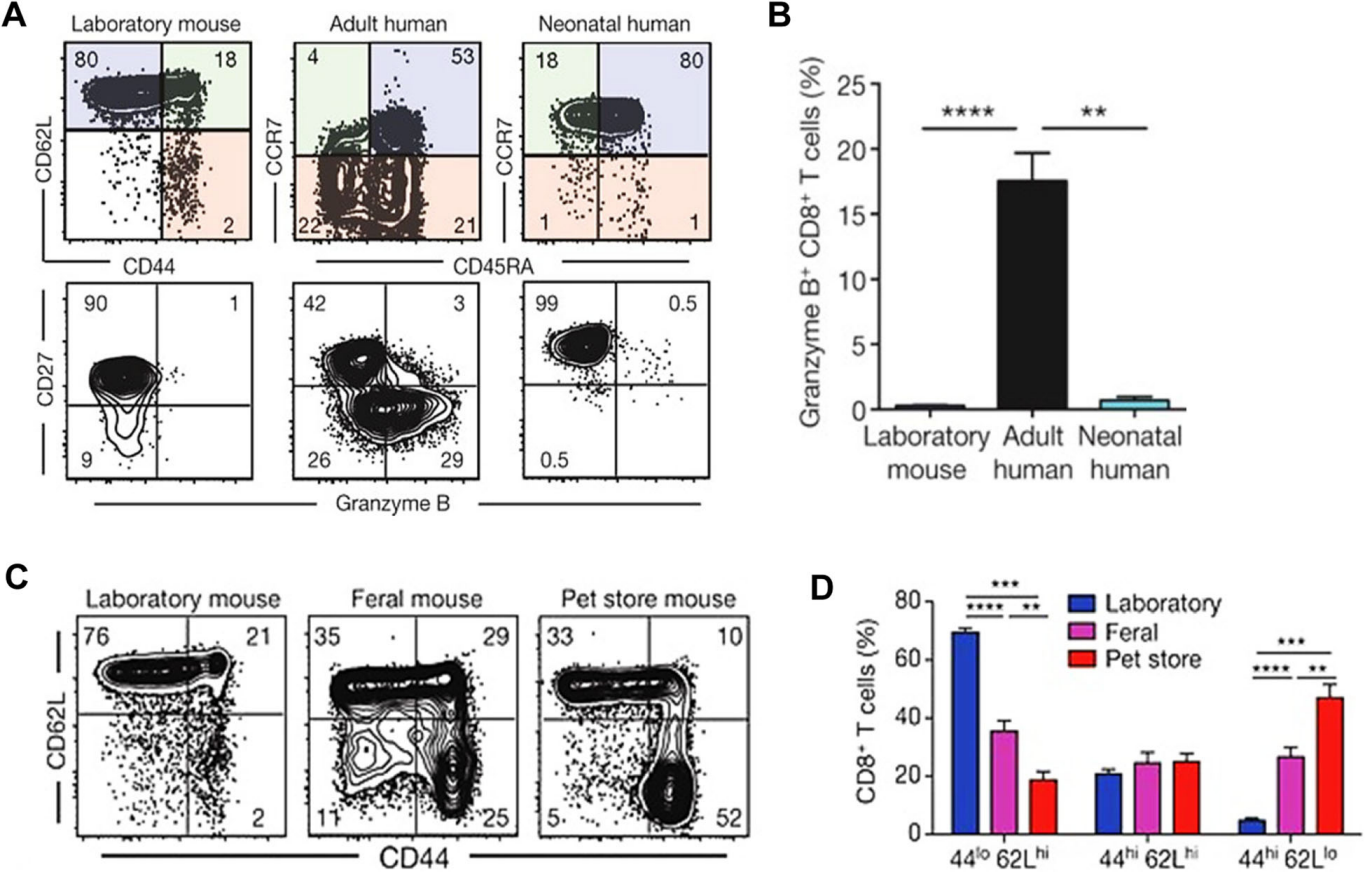
Mice housed under SPF/barrier conditions lack differentiated memory CD8^+^ T cell subsets compared with feral mice, pet store mice and adult humans. **a** Percentages of CD8^+^ T-cell phenotypes in laboratory/SPF mouse blood, adult human blood and human neonatal cord blood. The top panels are gated on CD3^+^/CD8^+^ cells with *naïve* T cells highlighted in blue, *central memory* T cells highlighted in green and *effector memory* T cells highlighted in red as defined by established lineage markers in each species. Bottom panels are gated on antigen-experienced subsets as defined in the green and red quadrants above). **b** Percentages of granzyme B^+^/CD8^+^ T-cells are much less in the antigen-experienced subsets of laboratory mice and neonatal humans when compared with adult humans. **c** and **d** Percentages of naïve CD8^+^ T cells (CD44^lo^/CD62L^hi^) in PBMCs from laboratory mice are much greater than those in PBMCs from feral and pet store mice whereas percentages of antigen-experienced C8^+^ T cells (CD44^hi^/CD62L^lo^) were significantly greater in feral and pet store mice when compared those in laboratory mice. Reproduced from [12] with permission

A recent study by Beura et al. demonstrated that feral (i.e. wild) or pet store-housed mice exhibited an immune cell composition similar to adult humans whereas SPF/barrier-housed mice possessed an immune system that was more akin to neonatal humans (Fig. 2a & b) [12]. These investigators found that SPF/barrier mice and neonatal humans have many fewer antigen-experienced, CD8^+^ memory/effector T cells when compared with feral or pet store-housed mice or adult humans (Fig. 2c & d). These and other analyses suggested that exposure of mice to the diverse spectrum of microorganisms that are normally encountered during the wild greatly influences the maturation and cellular composition of their immune system. Furthermore, these investigators showed that infection of SPF/barrier-housed C57BL/6 mice with the intracellular pathogenic bacteria *Listeria monocytogenes* (*LM*) had > 10,000-fold greater bacterial load than did pet store mice infected with same pathogen suggesting that exposure to diverse microbiota greatly increases resistance to pathogenic bacteria [12]. Although pet store mice do in fact possess a more diverse microbiota than SPF/barrier mice, it is likely to be less diverse than free-living feral mice and humans who are exposed to an even greater spectrum of viral, bacterial and parasitic micro-organisms.

**Fig. 3:**
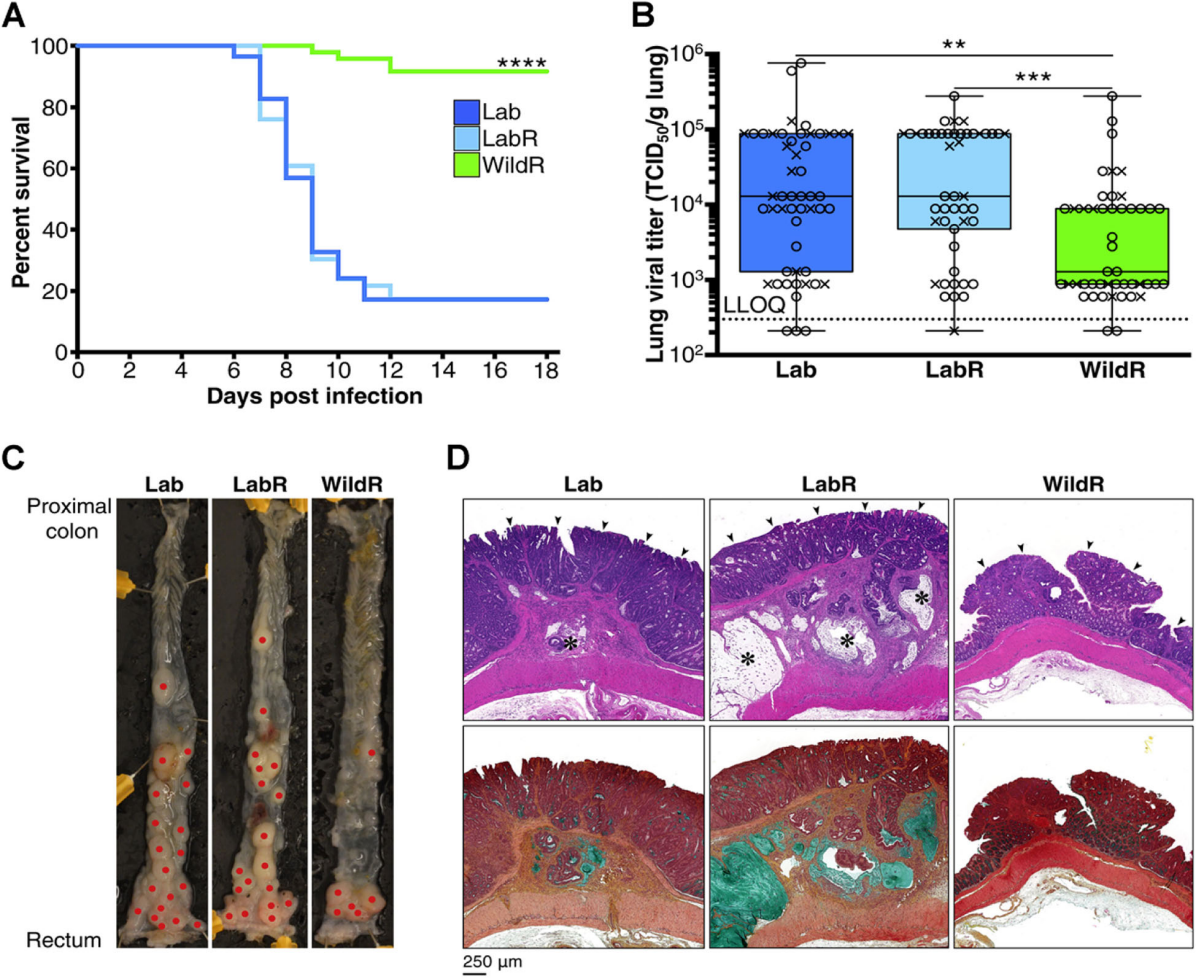
Colonization of germ free mice with microbiota obtained from feral mice protects against viral infection and inflammation-induced cancer. Pregnant, *germ free* C57BL/6 mice were colonized via oral gavage of gut microbiota obtained from feral mice (WildR mice) or from SPF C57BL/6 mice (LabR mice). Barrier-housed C57BL/6 mice (Lab mice) were used as controls to compare to WildR and LabR mice. **a** Male and female mice in all three groups of mice were infected (intranasally) with influenza A virus (IAV; Puerto Rico/8/1934 H1N1 strain). Approximately 92% of the WildR mice infected with IAV survived for 18 days post-infection whereas only 17% of the LabR and Lab mice were alive at 18 days post-infection. **b** Survival of WildR mice was associated with ∼10-fold lower titers of lung-residing IAV as well as significantly lower lung histopathology scores and inflammatory cytokine levels when compared with LabR and Lab mice. **c** Inflammation-induced colorectal cancer was induced in all three groups of mice via a single injection (*i.p.*) of the mutagen azoxymethane (10 mg/kg of body weight) followed by induction of colonic inflammation via oral (*ab libitum*) administration of dextran sodium sulfate (2–2.5%) in the drinking water. Representative images of dissected colons demonstrated greater numbers of colonic tumors (tumors indicated by red dots) in Lab and LabR mice when compared with WildR mice. **d** ***Top Panel:*** Representative histopathology (10x magnification) of H&E-stained sections of longitudinal colon tumors with arrows indicating well-differentiated adenocarcinoma in the mucosa. Histopathological analyses revealed that Lab and LabR developed tubular adenoma, well-differentiated tubular adenocarcinoma and mucinous carcinoma. Furthermore, mucinous carcinoma cells were found invading the submucosa and muscular layer where moderate-severe inflammation was also noted. Asterisks identify mucinous nodules. In contrast, WildR mice developed smaller numbers of colonic tumors, reduced tumor area/colon area, diminished tumor invasion and less inflammation when compared with the LabR and Lab mice that received the same treatment protocol. ***Bottom Panel:*** Movat’s staining of serial sections from the same tumors presented in the top panels clearly shows the presence of mucinous nodules (mucin stains green) containing mucinous carcinoma cells in submucosa and muscle layers of Lab and LabR but not WildR tumors. Reproduced from [15] with permission

Rosshart et al. recently tested the hypothesis that colonization of SPF/barrier-housed mice with intestinal microbiota obtained from feral mice would greatly influence immune responses to viral pathogens [15]. These investigators captured over 800 feral mice from horse barns located in several different regions of Maryland and Washington D.C. Of these, 98 mice were found to be *Mus musculus domesticus*, the feral species of mouse that is most closely related to common laboratory mice such as C57BL/6 mice. Surprising, despite being exposed to pathogens in the wild, a number of captured mice were found to be *pathogen-free* with no observable evidence of disease. Not surprisingly, Rosshart et al. determined that the intestinal microbiota of feral mice was significantly different from that of commercially available C57BL/6 mice [15]. Colonization of pregnant, *germ free* C57BL/6 mice was performed using daily oral gavage of microbiota (for 3 days) from pathogen-free feral mice or from SPF/barrier C57BL/6 microbiota. These mice were referred to as WildR and LabR mice, respectively. SPF/barrier-housed C57BL/6 mice (called Lab mice) were used to compare to WildR and LabR mice. All three groups of mice were then infected via *intranasal* administration of the mouse-adapted, influenza A virus (IAV; PuertoRico/8/1934 H1N1 strain). Surprisingly, 92% of the WildR mice infected with IAV survived for 18 days post-infection whereas only 17% of the LabR and Lab mice were alive at 18 days post-infection (Fig. 3a) [15]. Survival of WildR mice was associated with ∼10-fold lower titers of lung-residing IAV as well as significantly lower lung histopathology scores and inflammatory cytokine levels when compared with LabR and Lab mice (Fig. 3b). Overall, these exciting studies strongly suggested that colonization of laboratory mice with feral microbiota protects the host from a potentially lethal viral infection observed in SPF/barrier mice.

The studies described above are consistent with two other reports that characterized the differences in immune cell composition and responses in feral vs. laboratory mice infected with different pathogens. Abolins et al. foud that innate and adaptive immune cells in feral mice exist in a much higher state of activation when compared with barrier-housed laboratory mice [72]. Because feral but not SPF/barrier mice were found to be seropositive for several microbial, parasitic and myocoptes (mites) organisms, the authors suggested that alterations in the different immune cell populations and their responses were most likely driven by pathogen infection of these free-living rodents. These data agreed well with those reported by Reese et al. who found that sequential infection of SPF/barrier mice with three viral, bacterial and parasitic pathogens known to infect children, induced gene expression patterns in blood cells that were quite similar to those observed in pet store-housed mice and human adults [73].

##### Autoimmune and chronic inflammatory diseases

It is well known that the phenotype of different mouse models of autoimmune and chronic inflammatory diseases may be dramatically altered by changes in institution, housing conditions, and/or diet [74–77]. This is not surprising given the evidence demonstrating that the intestinal microbiota of mice may vary greatly among different animal care facilities and animal vendors [74, 75, 78–80]. Several laboratories including our own, have shown that the incidence and severity of chronic gut inflammation in different mouse models of inflammatory bowel disease is markedly altered by changing animal care facilities or by purposely altering the gut bacteria [81–87].

In addition to its effects on intestinal inflammation, alterations in gut bacteria have been found to greatly influence the onset and severity of joint inflammation. Using the K/BxN T cell receptor transgenic mouse (KBN) model of autoimmune arthritis [88], Wu et al. demonstrated that when KBN mice were housed under SPF/barrier conditions, they developed chronic and severe arthritis whereas housing these mice under germ free (GF) conditions exhibited greatly reduced disease [89]. In addition, when newborn KBN mice were continuously treated with vancomycin or ampicillin for several weeks, disease severity was markedly reduced suggesting that commensal microbiota were required for induction of disease [89]. Ivanov et al. had previously reported that a single bacterial species within the commensal flora called segmented filamentous bacteria (SFB) was capable of inducing the generation of intestinal lamina propria (I-LP) Th17 cells that are thought to play a role in the development of arthritis [79]. Thus, Wu et al. colonized GF mice with SFB to determine whether this one bacterial species could recapitulate the disease-producing effects of the SPF microbiota. Indeed, they found that colonization with SFB induced the generation of I-LP Th17 cells that produced robust autoimmune arthritis [89].

Similar findings were reported by Lee et al. who showed that while GF mice failed to develop EAE, GF mice colonized with SFB developed severe EAE [90]. These important studies demonstrated that a single group of commensal bacteria is capable of inducing autoimmune disease in different tissues via the generation of a specific disease-producing T cells. Although SFB are important in promoting inflammatory tissue damage in mouse models of arthritis and MS, other studies demonstrate that the presence of SFB may limit the development of other chronic inflammatory diseases such as diabetes. It has been known for more than 25 years that the incidence and severity of experimental autoimmune diabetes is markedly increased when nonobese diabetic (NOD) mice are housed under GF conditions suggesting that commensal bacteria may protect against autoimmune destruction of islet cells [76, 80]. Kriegel et al. took advantage of the fact that their NOD mouse colony was variably colonized with SFB to examine whether the presence or absence of SFB affected the incidence and/or severity of diabetes in males and females. They found that 91% of the SFB-negative (SFB-) females developed robust disease by week 30 whereas only 16% of the SFB-colonized (SFB+) females developed hyperglycemia [80]. In contrast, both SFB+ and SFB- males developed little or no disease with an overall incidence of < 20%. Furthermore, development of insulitis occurred in both SFB+ *and* SFB- females suggesting that the presence of SFB somehow interferes with the progression of islet inflammation to hyperglycemia.

##### Inflammation and tumorigenesis

Colorectal cancer (CRC) is one of the most common forms of cancer in modernized (i.e. westernized) societies that is predicted to increase over the next 20 years [91, 92]. It is becoming increasingly appreciated that changes in diet in modernized societies may contribute to the development of CRC by altering intestinal microbiota communities and immune responses that may promote gut inflammation [91, 93]. Because Rosshart et al. demonstrated that colonization of barrier mice with feral mouse microbiota protected these *WildR* mice from the lethal effects of the viral pathogen IAV (discussed above), this same group wished to determine whether WildR mice would also be protected in a mouse model of CRC [15]. To do this they used the well-characterized mutagen and inflammation model of CRC in which mice were injected (*i.p.*) with the mutagen azoxymethane followed by induction of colonic inflammation via oral administration of dextran sodium sulfate. This model exhibits chronic colitis that progresses to high grade dysplasia and development of CRC [15, 94]. They found that WildR mice developed smaller numbers of colonic tumors, reduced tumor area/colon area and diminished tumor and inflammatory cell invasion when compared with the LabR and Lab mice that received the same treatment protocol [15] (Fig. 3c).

**Fig. 4:**
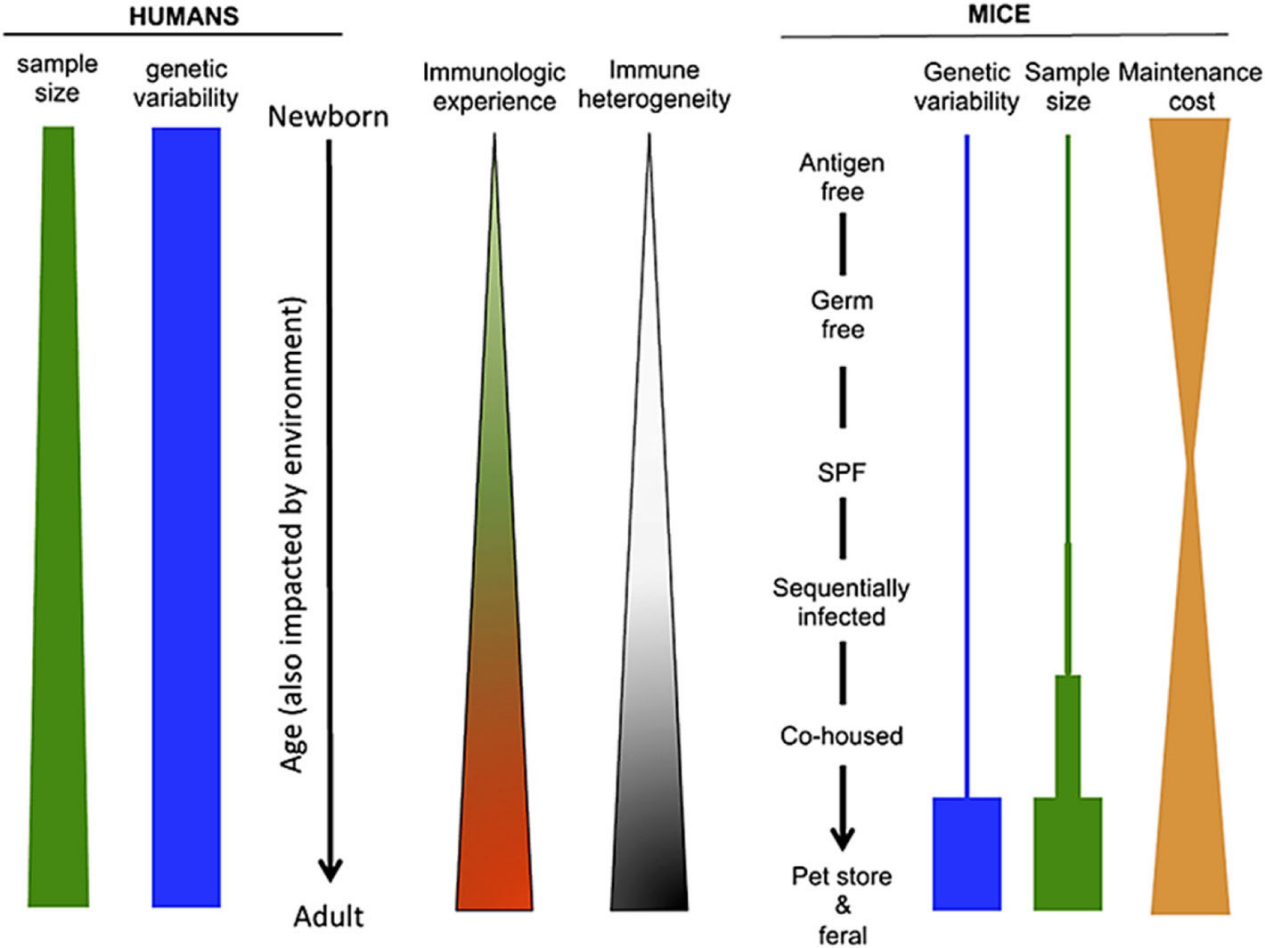
Genetic Variability, immunologic experiences and antigenic challenges in humans and mice. Humans are genetically more diverse and are subjected to highly variable, immunological experiences that shapes immune system development and function. Immunologic experiences in mice can be manipulated using different animal housing and husbandry protocols as well as exposure to a more diverse or natural microbiota. Reproduced from [71] with permission

There is no question that SPF/barrier housing has greatly enhanced reproducibility of immune responses and disease phenotypes. Nevertheless, the lack of exposure of lab mice to the spectrum of naturally occurring microorganisms, produces mice with an immune system that differs quite dramatically from feral mice and humans. It is becoming increasingly apparent immune system development in both mice and humans is shaped by exposure to a multitude of diverse immunological experiences that begin at birth (Fig. 4). Although there is a growing interest in the use of “dirty” mice to enhance translation of preclinical studies, the generation, use and housing of these mice may be quite difficult to implement. Because feral or pet store colonized mice would likely contain a plethora of viral, bacterial and parasitic pathogens that could quickly contaminate barrier facilities, dirty mice would have to be housed in a location that is physically separated from the barrier facility [71]. For example, dirty mice could be housed in a quarantine facility or in a location that does not house rodents. In order to prevent the spread of pathogens in these facilities, several protocols would have to be implemented such as the use of dedicated equipment for caging and for sanitation of cages and water bottles. In addition, it would be necessary to carefully control the movement and hygiene of animal care and laboratory workers as well as employ directional airflow to limit the spread of airborne pathogens outside of the animal room. Finally, the cost of housing these mice would undoubtedly be much greater than barrier housing. One intriguing protocol that creates mice that are colonized with natural/wild microbiota is called “rewilding”. Graham and coworkers recently reported that when lab mice are transferred to outdoor enclosures where they are exposed to the weather and microbiome that inhabits the soil and vegetation, they display maturation and differentiation of different T cell subsets, increased numbers of circulating granulocytes and changes in intestinal microbiota that are similar to those described above in feral and pet store mice [95–97]. When taken together, it is becoming clear that colonization of mice with diverse populations of naturally occurring microorganisms protects them against an environment that contains potentially lethal infectious microbes, inflammogens and carcinogens.

### Housing temperature

**Fig. 5:**
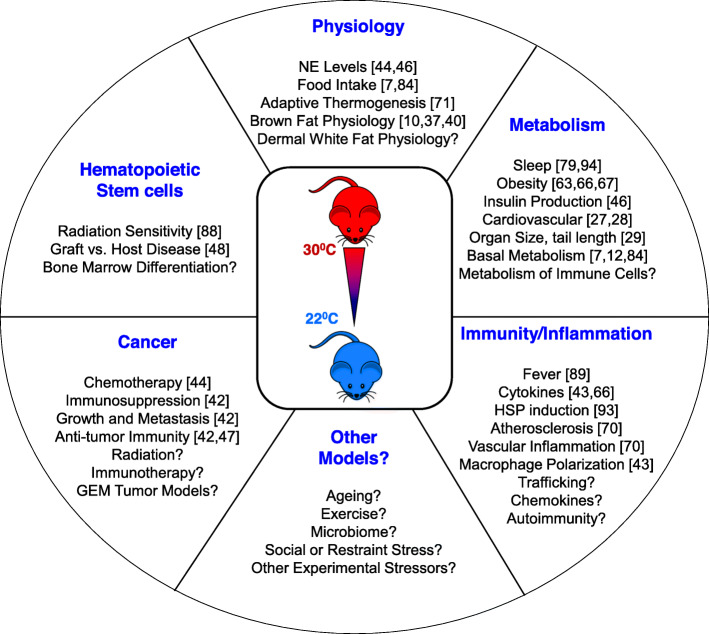
Housing temperature affects the physiology, immunology and immunopathogenesis pathogenesis of mouse models. This illustration depicts a number of different experimental settings and models in which outcomes differ in mice housed under standard animal care temperatures (∼22 °C) vs. housing at their thermoneutral temperature (∼30 °C). Reproduced from [23] with permission. References for the different studies in this figure appear in reference [23]

Another environmental parameter that has received a great deal of attention over the past several years is the influence of housing temperature on murine physiology and immunology. Virtually all animal care facilities in the U.S. house mice at 20–24 °C, a temperature range that is comfortable for human caregivers. However, this temperature range induces mild but *chronic* cold stress in these rodents as their thermoneutral temperature (TNT) is 30–32 °C [22–24, 98–100]. Murine TNT is defined as “the temperature range within which the heat produced as a byproduct of normal metabolism alone, combined with blood flow movements from the core to the surface of the body, enables an animal to maintain a normal core body temperature of ~37°C” [22, 23]. Housing mice at standard animal care temperatures (ST; 20–24 °C) is known to increase their resting heart rate and basal metabolic rate when compared with mice housed at their TNT [24]. In addition, housing mice at ST activates the sympathetic nervous system (SNS) resulting in the release of the catecholamines epinephrine (Epi) and norepinephrine (NE) as well as activates the hypothalamic-pituitary-adrenal axis (HPA) to induce production of glucocorticoids [22]. Because catecholamine and glucocorticoid receptors are found on nearly all cells within the body, cold stress-induced activation of the SNS and HPA produces marked alterations in the cardiovascular, skeletal-muscular and immune systems [23, 101]. (Fig. 5). Furthermore, it is becoming increasing appreciated that cold stress may exert profound effects on intestinal homeostasis and microbial composition [22, 102, 103].

This inconvenient reality has prompted investigators to examine how housing temperature may influence immune responses in mice. It is well known that primary lymphoid tissue (thymus, bone marrow) as well as the spleen, lymph nodes and mucosa-associated lymphoid tissue are innervated by sympathetic nerves suggesting that NE, the major neurotransmitter released by sympathetic nerves, is involved in modulating immune responses [104–106]. Indeed, it has been shown that NE-induced β2 adrenergic receptor (β2-AR) signaling in T and B cells, myeloid cells and dendritic cells (DCs) modulates antigen-processing and presentation as well as T cell activation, differentiation and recirculation [104, 106–113]. A recent study by Araujo et al. demonstrated that the SNS limits the development of MOG_35–53_/CFA-induced experimental autoimmune encephalomyelitis (EAE) in mice via β2-AR signaling in CD4^+^ T cells that constrains the generation of encephalogenic effector cells [107]. In contrast, when β2-AR-deficient (β2-AR^*−/−*^) mice were immunized with MOG_35–53_/CFA, they developed more severe disease when compared with wild type mice. Taken together, these data clearly demonstrate that the SNS plays an important role in regulating CNS autoimmunity. It will be interesting to assess the role of the SNS in mouse models EAE or other autoimmune disease housed at their TNT. The following section presents a brief overview of the effects of housing temperature on infection, immunity and inflammation. The reader is referred to the following references that summarize the role of housing temperature in mouse models of cancer and antitumor immunity [22, 23, 112, 114, 115]. In addition, investigators have shown that cold stress may exert profound effects on intestinal homeostasis and microbial composition [22, 102, 103].

#### Infection and immunity

As pointed out above, β2-AR are found on all myeloid cells as well as T and B cells. Stress induced release of NE and its engagement with the β2-AR modulates immune responses to infectious microorganisms [21]. Although relatively few studies have directly assessed the effects of housing temperature on infection and immunity, there are numerous studies demonstrating that β2-AR signaling is generally immunosuppressive and results in increased susceptibility of the mice to pathogen infection (the reader is referred to these reviews: [21, 104, 106, 114]. Using selective β2-AR agonists and antagonists or genetic ablation of the receptor, several laboratories have described the role that β2-AR signaling pathways play in infection with different bacterial (*L. monocytogenes, P. aeruginosa, S. typhimurium, K. pneumonia, E. coli)* or viral pathogens (Cytomegalovirus, Herpes simplex, Influenza, Vesicular stomatitis) [21, 114]. Cold stress-induced immune suppression has been suggested to account for the high mortality rate and lack of clinical translation of mouse models of infectious disease [24, 116–118]. In a recent study, Rubin directly compared different immune responses to the *Francisella tularensis (Ft)* live vaccine strain in mice housed at ST (22 °C) to mice housed at temperatures approximating their TNT (28 °C). Rubin demonstrated that when mice were housed at their TNT they exhibited increased antigen-specific T-cell responses compared with mice housed at 22 °C [116]. In addition, Rubin found that intranasal challenge of *Ft* to immunized mice housed at 22 °C was consistently fatal whereas immunized mice housed near their TNT (28 °C) survived the same intranasal challenge. When taken together, this study demonstrates that mice housed below their TNT exhibit diminished T cell responses to this intracellular pathogen resulting in animal death. Furthermore, this study as well as others referenced above suggest that housing mice at their TNT may help to improve the translation of data obtained from mouse models of infectious disease and vaccine development.

#### Inflammation

##### Obesity, metabolic inflammation and atherosclerosis

Excessive ingestion of lipid-laden foods is known to induce low grade but chronic inflammation that is thought to play a role in the pathogenesis of different metabolic diseases such as obesity, atherosclerosis, chronic liver disease, type 2 diabetes and cancer [119, 120]. Indeed, obesity has become an international epidemic that is the second most preventable cause of death in modernized societies [121]. One life-threatening disease associated with obesity is atherosclerosis. This cardiovascular disease is the leading cause of death of men and women in the U.S. [69]. Histopathological examination of atherosclerotic vessels reveals the presence of cholesterol and immune cells in the arterial wall that ultimately progresses to plaque formation and occlusion of blood flow. Defining the immuno-pathogenic mechanisms responsible for obesity-induced vascular plaque formation and progression has been difficult to model in mice since wild type mice do not develop atherosclerosis when placed on a high fat/high cholesterol Western diet (WD). Much of what we have learned has come from the use of genetically-engineered mice that lack the low-density lipoprotein receptor (LDR^−/−^) or apolipoprotein E (ApoE^−/−^). Data obtained from studies using these mutant mice have been important in revealing the role of lipid accumulation and immune cell infiltration in hyperlipidemia-induced vascular inflammation and plaque formation; however, neither mouse model exhibits lipid profiles and vascular pathology that are identical to patients with atherosclerosis [122, 123].

Differences between these models of atherosclerosis and human disease have prompted investigators to develop additional mouse models that more closely recapitulate the pathophysiology of this vascular occlusive disease. Recent studies demonstrating that housing mice at their TNT affects cardiovascular physiology, inflammation and metabolism have motivated investigators to examine how housing temperature may influence the development and progression of obesity, metabolic inflammation and atherosclerosis in mice fed a high fat diet (HFD). Giles et al. reported that feeding a WD to C57BL/6 mice housed at their TNT (30 °C) enhanced weight gain and fat mass when compared with mice fed a WD diet while housed at ST (22 °C) [124]. In addition, they demonstrated that TNT housing in combination with WD induced mild atherosclerosis that was associated with increases in serum concentrations of total cholesterol and LDL, aortic plaque formation, and greater immune cell infiltration into the vascular lesions when compared to mice fed a WD and housed at ST [124]. Furthermore, both Giles et al. [124] and Tian et al. [119] demonstrated that feeding a WD to ApoE^−/−^ mice housed at their TNT greatly enhanced the development of obesity as well as accelerated the onset and severity of atherosclerosis when compared with their ST controls. Thus, both studies demonstrate that housing temperature plays an important role in the development of metabolic inflammation, obesity and atherosclerosis. The development of increased weight gain and adiposity at TNT has also been reported by Stemmer et al. in T cell-deficient, C57BL/6 nude (nu/nu) mice [125]. These results have important implications for investigators who wish to model the effects of obesity on human tumor development in vivo*.* Obesity has been linked to the development of a variety of different types of cancers; however, the vast majority of human tumor xenograft studies have been performed using obesity-resistant BALB/c nude mice. Thus, the data reported by Stemmer et al. may provide a novel mouse model for assessing the impact of obesity on human tumor growth in vivo.

##### Nonalcoholic fatty liver disease

Another, potentially life-threatening condition that is strongly associated with obesity is nonalcoholic fatty liver disease (NAFLD). This chronic condition is characterized by the accumulation of excess fat in the liver of individuals who drink little or no alcohol. The most common form of NAFLD is a relatively innocuous condition referred to as nonalcoholic fatty liver (NAFL). Although fat accumulation within hepatocytes is alarming, liver function does not appear to be impaired by this condition. Some individuals with NAFL may go on to develop a more serious condition called nonalcoholic steatohepatitis (NASH). The livers of these individuals exhibit fat accumulation, inflammatory cell infiltration and varied levels of fibrosis. If left untreated, NASH may ultimately progress to cirrhosis and hepatocellular carcinoma [126–129]. Although current mouse models of NAFLD have been important in delineating certain aspects of disease pathogenesis, they do not fully recapitulate human disease. For example, mouse models are restricted to the use of male mice since females do not develop high fat diet (HFD)-induced obesity and NAFLD. In addition, mouse models of NAFLD do not develop the fibrosis (called “bridging fibrosis”) that is observed in humans. Therefore, investigators are attempting to develop mouse models that more closely recapitulate NASH in both male and female mice. Giles et al. recently reported that feeding male C57BL/6 mice with a HFD housed at their TNT (30 °C) induced an acceleration in weight gain, increased liver weight and steatosis and an increase in liver immune cell infiltration when compared with mice that were fed a HFD and housed at ST (22 °C) [126]. In addition, they found that HFD and TNT housing enhanced expression of chemokine and fibrosis genes as well as increased infiltration of macrophages when compared with HFD/ST mice. These TNT-induced alterations were associated with increased hepatocellular injury as determined by an increase in serum levels of alanine transaminase. Interestingly, the authors did not observe overt fibrosis in these mice which is not surprising given the well-known fact that C57BL/6 mice are resistant to developing hepatic fibrosis [126].

In a second series of studies, Giles et al. used male AKR mice, an inbred strain that has been shown to develop marked obesity and NAFLD when fed a HFD at ST (22 °C). When these mice were housed at their TNT (30 °C) and fed a HFD, they developed many of the same features and histopathological characteristics of NAFLD as did C57BL/6 mice; however, HFD/TNT AKR mice developed hepatic fibrosis suggesting that these mice may be better suited to model human NASH [126]. As noted above, the vast majority of preclinical studies have been performed using male mice since a HFD does not induce obesity and NAFLD in females. However, the prevalence of NAFLD in male and female humans is virtually the same [126]. Therefore, Giles et al. assessed the development of NAFLD in female C57BL/6 mice when housed at their TNT. They found that when female mice were housed at their TNT and fed a HFD, they displayed remarkable increases in body weight, liver weight, tissue adiposity and hepatic steatosis when compared with female HFD/ST mice [126]. Similar to male HFD/TNT mice, females housed under the same NAFLD-producing conditions exhibited increased expression of genes associated with fibrosis and augmented hepatocellular injury but no overt fibrosis. Nevertheless, the development of these novel mouse models may allow investigators to identify novel the immuno-pathogenic mechanisms responsible for the development and progression of NAFLD in both sexes. Another interesting aspect of NAFLD development in both mice and humans is the relationship among intestinal dysbiosis, increased intestinal permeability and hepatocellular damage. In their recent study, Giles et al. also observed greater increased numbers of live bacteria in the livers of HFD/TNT mice that appeared to correlate with dysbiosis and increased intestinal permeability when compared with their HFD/ST counterparts. In fact, these investigators demonstrated that the bacterial composition of the dysbiotic microbiome in HFD/TNT mice was similar to that observed in humans with NASH [126]. Administration of neomycin and polymixin B sulfate reduced intestinal permeability, hepatic inflammation scores and liver damage when compared with obese, antibiotic-treated HFD/ST mice. Taken together, these data suggest that exacerbation of NAFLD in HFD/TNT mice may be mediated by alterations in intestinal bacterial composition.

##### Acute graft vs. host disease

**Fig. 6:**
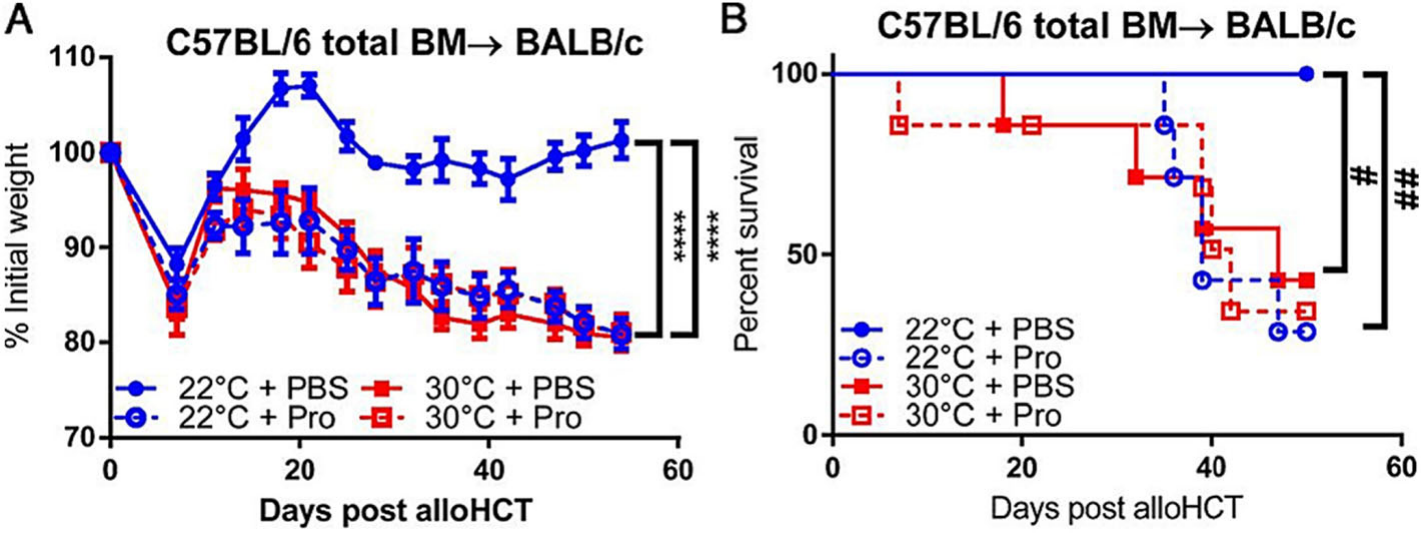
Housing mice at their thermoneutral body temperature (30 °C) exacerbates the development of acute graft vs. host disease (aGVHD). Disease was induced by adoptive transfer of allogeneic C57BL/6 bone marrow (BM) alone into lethally-irradiated BALB/c recipients. Mice were housed at either 22 °C or 30 °C and monitored for signs of GVHD such as weight loss (**a**) and survival (**b**). Reproduced from [98] with permission

Allogeneic hematopoietic stem cell transplantation (HSCT) is a potentially life-saving treatment for certain blood cancers, autoimmune diseases or hematologic disorders (sickle cell disease, Fanconi anemia) [130]. Although allogeneic HSCT is a potential cure for these diseases, ~ 50% of patients receiving this treatment will develop a potentially life-threatening, multi-organ inflammatory condition called acute graft versus host disease (aGVHD) [131, 132]. Clinically, inflammation may occur in the gastrointestinal (GI) tract, skin, liver, lungs, bone marrow and lymphoid tissue [131–137]. The immuno-pathogenesis of aGVHD has not been completely defined; however, experimental and clinical studies demonstrate that donor CD4^+^ and/or CD8^+^ T cells are the major effector cells responsible for mediating inflammatory tissue injury in the different target tissues [132]. The large majority of mouse models of aGVHD use lethal, whole body irradiation to ablate the immune system prior to engraftment of allogeneic bone marrow (BM) that is supplemented with allogeneic T cells [138]. Adoptive transfer of BM alone serves as the control group for these studies since recipients do not develop aGVHD when housed at standard animal housing temperatures of 20–22 °C. It should be noted that this contrasts with clinical HSCT in which great care is taken to eliminate all T cells from the donor graft as the presence of even small numbers of residual, graft-associated T cells may induce fulminant disease [132]. In a landmark study using the conventional mouse model of aGVHD described above, Leigh et al. demonstrated that aGVHD does develop in lethally-irradiated recipients engrafted with allogeneic BM alone, provided that the mice are housed at their TNT of 30 °C [98] (Fig. 6). In contrast, these investigators confirmed numerous other studies demonstrating that aGVHD fails to develop in BM-engrafted mice housed at ST of 22 °C. They found that ST-mediated suppression of disease was due to cold stress-induced β2-AR signaling by NE [98]. Treatment of BM-engrafted mice housed at 22 °C with a selective β2-AR antagonist reversed the cold stress-induced, NE-mediated suppression of aGVHD such that these mice developed disease that was similar to BM-engrafted mice housed at 30 °C [98]. Conversely, when BM-engrafted mice were housed at 30 °C and then treated with a selective β2-AR agonist, little or no disease was observed demonstrating that β2-AR signaling is crucial for suppression of aGVHD. A recent study by Mohammadpour et al. extended these findings using different models of allogeneic and xenogeneic models of HSCT. They found that adoptive transfer of allogeneic, β2-AR deficient (β2-AR^−/−^) CD4^+^ T cells (with BM) into lethally irradiated recipients induced more severe aGVHD than did engraftment with wild type (WT) allogeneic T cells [139]. The exacerbation of disease in mice engrafted with β2-AR^−/−^ CD4^+^ T cells was associated with increased numbers Th1 effector cells whereas transfer of WT T cells resulted in increases in Tregs, Th2 cells and myeloid derived suppressor cells [139]. Taken together, these data suggest that selective β2-AR agonists may prove useful in attenuating aGVHD.

##### Asthma

Approximately 70% of patients suffering from asthma have reported difficulty breathing in cold air during the winter months [140]. Although investigators and clinicians have suggested that inhalation of cold air may exacerbate asthma by inducing epithelial cell injury as well as immune cell infiltration and mast cell activation in the airways [141–143], few mechanistic studies have been reported to address whether ambient air temperature may alter the onset and/or severity of this chronic respiratory disease. A recent study by Liao et al. compared disease symptoms and immune responses in asthmatic mice housed at ST (20 °C) or at TNT (30 °C). Airway inflammation was induced using a well characterized mouse model of acute asthma induced by intraperitoneal sensitization of the mice with ovalbumin (OVA) followed by intratracheal challenge with aerosolized OVA in saline. This protocol induces airway hyper-responsiveness, eosinophil infiltration into the bronchi and overproduction of mucus [144]. These investigators found that when asthmatic mice were housed at their TNT, inflammatory cell numbers, IL-4 and IL-13 levels were all significantly reduced in the bronchoalveolar lavage fluid as were serum levels of IgE and airway hyper-responsiveness in TNT vs ST mice. These data provide some of the first evidence that housing temperature may influence the onset and/or severity of acute bronchial inflammation and dysfunction.

## Conclusions

The use of genetically standardized mice and housing conditions have greatly advanced our fundamental understanding of innate and adaptive immunity as well as increased the reproducibility of animal studies. Nevertheless, translation of promising therapeutic strategies observed in mouse models of infectious and inflammatory diseases to patient treatment has been disappointing. The reasons for the poor bench-to-beside transition have not been definitely defined; however, emerging evidence suggests that exposure of genetically constrained mice to pathogen-free microbiota at sub-optimal housing temperatures may impair maturation of the immune system thereby altering the animal’s immune responses to infectious microorganisms and inflammatory mediators. We propose that by using genetically diverse mice housed under more immunologically- and environmentally-relevant conditions may improve the chance of identifying new and more potent therapeutics to treat human disease.

## Data Availability

The datasets used and/or analyzed during the current study are available from the corresponding author on reasonable request.
